# Optimal Management of Patients with Phlegmonous Esophagitis: A Systematic Review and Meta-Analysis

**DOI:** 10.3390/jcm12227147

**Published:** 2023-11-17

**Authors:** Dae Hyun Jin, Wongi Woo, Jimin Lee, Duk Hwan Moon, Sungsoo Lee

**Affiliations:** Department of Thoracic and Cardiovascular Surgery, Gangnam Severance Hospital, Yonsei University College of Medicine, Seoul 03722, Republic of Korea; dhjin1105@gmail.com (D.H.J.); woopendo@gmail.com (W.W.); jsk12@yuhs.ac (J.L.); pupupuck@yuhs.ac (D.H.M.)

**Keywords:** phlegmonous esophagitis, prognosis, systematic review

## Abstract

Goals: To assess the characteristics and prognosis-influencing factors of phlegmonous esophagitis, a rare condition marked by suppurative inflammation of the esophageal submucosa and muscular layers. Background: Effective management strategies for phlegmonous esophagitis are lacking. This study aims to systematically review cases to better understand the disease’s features and prognostic determinants. Method: A systematic search was performed using PubMed/MEDLINE and Google Scholar from inception to 20 April 2023. Phlegmonous esophagitis case reports and studies with patient information were included; clinical manifestations, laboratory results, imaging findings, other diagnostic findings, and outcomes were analyzed. A pooled analysis was performed to investigate mortality-related risk factors. Results: A total of 35 cases of phlegmonous esophagitis were selected from 31 case reports and 2 case series (median age, 57.0 years; male, 54.3%). The patients presented various clinical symptoms, with neck-to-epigastric pain and dysphagia being the most common. Comorbid diabetes mellitus was a major predisposing factor; one-third of the patients had no previous medical history. Computed tomography (CT) and endoscopic examinations were predominantly used for phlegmonous esophagitis diagnosis. Radiological findings showed that the upper and middle esophagus were most frequently involved. Treatments included administration of broad-spectrum antibiotics and drainage via endoscopy or surgery. There were three cases of mortality, and non-survivors tended to have shorter hospital stays, indicating rapid disease progression. In logistic regression, thoracic surgery was a significant mortality-related risk factor (odds ratio, 19.30; 95% confidence interval, 1.33–282.00, *p* = 0.03). Conclusion: Advancements in CT and endoscopy have led to less-invasive diagnostic and treatment methods for phlegmonous esophagitis. Endoscopic localized abscess treatment is associated with positive outcomes.

## 1. Introduction

Phlegmonous enteritis is an infection characterized by purulent inflammation of the submucosa and muscularis of the gastrointestinal tract with an unaffected mucosa. This condition was initially reported by Cruveilhier in the early 18th century. Although many studies have reported on phlegmonous gastritis [1], phlegmonous esophagitis remains poorly understood, despite its life-threatening potential [1,2,3,4,5,6,7,8,9,10,11,12,13,14,15,16,17,18,19,20,21,22,23,24,25,26,27,28,29,30,31,32,33].

Patients with phlegmonous esophagitis exhibit a broad spectrum of clinical manifestations that range from fever, sore throat, and chest pain to shock [2,3,4,5,6]. The reported predisposing factors include immunosuppression, alcoholism, uncontrolled diabetes mellitus, old age, malnutrition with low albumin levels, low socioeconomic status, and tumor burden [4]. The diagnosis of phlegmonous esophagitis was initially mainly reliant on surgery or autopsy. However, with advancements in diagnostic imaging, computed tomography (CT) and endoscopy have emerged as effective diagnostic tools [3,7,8]. Yun et al. reported the radiological characteristics of phlegmonous esophagitis and presented a flow chart for its differential diagnosis and treatment [8]. Regarding treatment regimens, broad-spectrum antibiotics and surgery are typically used; however, a recently developed endoscopic approach has shown favorable outcomes. Kim et al. reported that endoscopic drainage, in addition to traditional open surgery, is a feasible treatment option for phlegmonous esophagitis [9]. Several studies have described the benefits of jejunostomy or gastrostomy for early enteral feeding, resulting in better clinical outcomes [3,4,7,10,11]. Without early diagnosis and proper treatment, phlegmonous esophagitis can lead to many complications, such as peritonitis, mediastinitis, empyema, perforation, or sepsis—and even mortality.

Karimata et al. conducted a review of 13 cases of phlegmonous esophagitis [3]. However, the limited number of reported cases has hindered the understanding of this disease. Herein, we analyzed 35 cases from the published literature to conduct a comprehensive systematic review and identify prognostic factors related to disease outcomes.

## 2. Materials and Methods

### 2.1. Search Strategy and Selection Criteria

This review was performed in accordance with the Preferred Reporting Items for Systematic Reviews and Meta-Analyses Protocols (PRISMA-P, Supplemental Table S1). Additional Synthesis Without Meta-analysis (SWiM) was also used to evaluate this study, as it represents a less common type of meta-analysis that incorporates case reports/series (Supplemental Table S2). The requirement for ethical approval by the Institutional Review Board was waived due to the study design being based on published medical literature.

Herein, the inclusion criterion for reported cases was the definitive diagnosis of phlegmonous esophagitis or phlegmonous esophagogastritis using surgical or radiological methods. Patients with obvious secondary infections resulting from direct perforations caused by foreign bodies or trauma were excluded. Data was not excluded in cases where there was a possibility of perforation due to a foreign body, but a clear causal relationship could not be established through examination. Additionally, review articles, abstracts, letters to the editor, and articles that did not contain sufficient information regarding patient characteristics or outcomes were excluded. For instance, the paper was excluded if it did not contain independent data for individual patients such as demographics, laboratory results, radiologic findings, or clinical outcome. Articles in English, Korean, and Japanese were collected.

The PubMed and Google Scholar databases were searched to identify articles published until 20 April 2023, regarding patients with phlegmonous esophagitis or esophagogastritis. Detailed search strategies/terms and results can be found in Supplemental Figure S1 and Section 3.

### 2.2. Data Collection

Data regarding the demographic and clinical characteristics of patients, such as age, sex, medical history, symptoms, laboratory results, radiological findings, treatment methods, and clinical outcomes, including length of hospitalization, complications, and mortality, were collected and sorted. Non-existing or ambiguous data were excluded from the analysis. D.H.J. and W.W. screened literature and collected data. Any disagreements between the reviewers regarding any study were resolved through discussion until a consensus was reached. When there was disagreement, it was supervised by S.L. 

### 2.3. Statistical Analysis

The Statistical Package for the Social Sciences (SPSS) for Windows, version 25.0 (SPSS Inc., IBM Corp., Armonk, NY, USA), and R, version 4.0.4 (R Core Team, R Foundation for Statistical Computing, Vienna, Austria; https://www.R-project.org/ accessed on 6 June 2023.) were used for statistical analysis. Continuous variables are presented as medians and interquartile ranges (IQRs), and categorical variables are presented as numbers and percentages. Continuous variables were compared using the Mann–Whitney *U*-test, and categorical variables were compared using Fisher’s exact test. Logistic regression analyses were performed to identify independent risk factors for mortality. Variables with a *p*-value ≤ 0.05 were considered significant.

## 3. Results

### 3.1. Systematic Search and Selection of Studies

A total of 115 articles regarding keyword phlegmonous esophagitis were identified using PubMed. Additional searches using the keywords “phlegmonous esophagogastritis”, OR “phlegmonous gastroesophagitis”, OR “phlegmonous enteritis” showed 24 articles in advanced searching and returned one or more studies that satisfied the inclusion criterion, apart from the duplicate results already found. A total of 23 papers in PubMed that met the inclusion criteria were included. A similar search was conducted on Google Scholar (“phlegmonous esophagitis” OR “phlegmonous esophagogastritis” OR “phlegmonous gastroesophagitis,” language filter: English/Korean/Japanese), which resulted in 172 studies. Among these, 10 additional studies were identified that were not duplicates of the results obtained in the PubMed search. The abstracts and full texts of the articles were reviewed, and all 33 studies (31 case reports and 2 case series) met our inclusion criteria. The included studies and their abstracts are listed in Supplemental Table S3.

### 3.2. General Demographics and Clinical Manifestations

Table 1 shows the patients’ characteristics. The median age of the patients was 57.0 (IQR, 48.0–66.0) years, and approximately half (19/35, 54.3%) were males. The patients had histories of diabetes mellitus (13/30, 43.3%), hypertension (6/30, 20.0%), alcoholism (5/31, 16.1%), cancer (2/30, 6.7%), chronic obstructive pulmonary disease (1/30, 3.3%), and hypothyroidism (1/30, 3.3%). Common symptoms included neck-to-epigastric pain (32/33, 97.0%), obstruction symptoms such as dysphagia or foreign body sensation (19/33, 57.6%), fever (18/32, 56.2%), nausea and/or vomiting (8/33, 24.2%), odynophagia (8/33, 24.2%), upper respiratory infection symptoms (5/33, 15.2%), gastrointestinal bleeding (5/33, 15.2%), and oropharyngeal infection (2/33, 6.1%). Patients had a median fever of 38.3 °C and tachypnea at the time of initial presentation.

Laboratory results showed that over half of the patients had leukocytosis (18/29, 62.1%), and a small number of patients presented with leukopenia (3/29, 10.3%). Most patients demonstrated elevated C-reactive protein levels (median, 19.3; IQR, 12.6–31.6 mg/dL), and approximately 30% of patients exhibited an accompanying increase in aspartate aminotransferase (median, 58.0; IQR, 18.0–76.0 IU/L), alanine transaminase (median, 50.5; IQR, 24.8–95.8 IU/L), and creatinine (median, 0.8; IQR, 0.8–1.2 mg/dL) levels. Pathogen identification via culture was performed in 21 of 24 cases; the causal pathogens are listed in Supplemental Table S3. Klebsiella pneumoniae (8/24, 33.3%) and Streptococcus spp. (6/24, 25.0%) were the most frequently identified infectious agents.

### 3.3. Radiologic Findings of Patients with Phlegmonous Esophagitis

The radiologic findings of the patients with phlegmonous esophagitis and esophagogastritis are shown in Table 1. Chest radiography revealed mediastinal widening (4/9, 44.4%), pleural effusion (5/10, 50.0%), and other findings (4/9, 44.4%). Approximately 90% (32/35) of patients underwent a chest CT, which revealed abscess formation, diffuse fluid collection/hypodense lesion in the esophagus, diffuse esophageal wall thickening, pleural effusion, the presence of air bubbles, mediastinal involvement, and obvious esophageal perforation. In the entire cohort, 29 (82.8%) patients underwent esophagogastroduodenoscopy, with reported ulcer/erosion, esophageal perforation, obstruction, and abscess formation. As regards radiological findings, the middle (32/35, 91.4%) and upper (31/34, 91.2%) esophagi were the most frequently involved sites. The proportions of cases demonstrating radiological or surgical evidence of invasion were as follows: lower esophagus (30/35, 85.7%), lungs (18/35, 42.9%), mediastinum (10/33, 30.3%), and pharynx (8/33, 24.2%).

### 3.4. Treatment and Clinical Outcome of Patients with Phlegmonous Esophagitis

Table 2 shows the clinical course and outcomes of the included patients. A total of fourteen patients (14/35, 40.0%) received antibiotics and conservative treatment, while 21 patients (21/35, 60.0%) underwent pus drainage procedures. In two cases where drainage was not pursued, spontaneous natural drainage occurred from the esophageal mucosal perforation site. Various drainage methods were used, including endoscopic drainage (6/35, 17.1%), thoracic surgery (5/35, 14.3%), and other methods (10/35, 28.6%) such as the gastric surgical approach or cervical drainage. The median interval between admission and the first drainage intervention was 2.0 (IQR, 1.3–5.8) days. In 25.7% of cases (9/35), additional pus drainage was performed either endoscopically (4/9, 44.4%) or surgically (6/10, 60.0%), which was necessary because of clinical deterioration in 75.0% of cases (6/8) or the presence of residual pus in 25.0% of cases (2/8). The median time from admission to the first oral intake was 36.0 (IQR, 14.0–74.0) days. In six cases (6/6, 100.0%), a jejunostomy or gastrostomy was performed for early enteral feeding.

The median length of hospital stay was 37.0 (IQR, 17.8–69.0) days. Only three cases of mortality were recorded, all among men. Common complications during hospitalization included esophageal stricture (9/24, 37.5%), disseminated intravascular coagulopathy (4/24, 16.7%), septic shock (4/25, 16.0%), gastrointestinal inflammation or scarring (2/23, 8.7%), and acute kidney injury (1/22, 4.5%).

### 3.5. Risk Factor for Mortality among Patients with Phlegmonous Esophagitis

When we compared survivors and non-survivors (Table 1 and Table 2), we found statistically significant differences in systolic blood pressure (120.0 vs. 99.0, *p* = 0.047), creatinine levels (0.8 vs. 2.1, *p* = 0.034), and the presence of pleural effusion on CT (100% vs. 0.0%, *p* = 0.067). Regarding clinical outcomes, the length of hospital stay was shorter in non-survivors (52.0 vs. 5.0 days, *p* = 0.006), which implied a rapid deterioration of the patients’ condition. Notably, more patients in the non-survivor group underwent thoracic surgery (9.4% vs. 66.7%, *p* = 0.047).

Logistic regression analysis to identify risk factors for in-hospital mortality revealed that pus drainage through thoracic surgery was a significant risk factor for mortality (odds ratio, 19.30; 95% confidence interval, 1.33–282.00; *p* = 0.03), as shown in Table 3.

## 4. Discussion

Acute phlegmonous esophagitis and esophagogastritis are rare, life-threatening diseases. During their clinical course, rapid deterioration is often observed, emphasizing the need for prompt diagnosis and appropriate treatment. In this systematic review, we collated information from 35 cases of phlegmonous esophagitis to understand the clinical manifestations, diagnostic methods, treatment options, and possible complications associated with this rare disease. Furthermore, we aimed to identify the risk factors associated with mortality.

Contrary to concerns regarding the high mortality rate of phlegmonous esophagitis reported in many previous studies, our review revealed a low occurrence of mortality (3/35, 8.6%). This was not considered significantly higher compared with that of phlegmonous gastritis, a common type of phlegmonous enteritis that has a mortality rate of approximately 40% following late diagnosis [34]. There are several possible explanations for this. First, advancements in diagnostic technologies, especially CT, have led to early identification, before clinical deterioration can occur. Previously, the diagnosis of phlegmonous esophagitis was challenging as no pathognomonic signs or clear diagnostic test results could be obtained. Simple radiography or ultrasonography could provide assistive information, such as mediastinal widening, empyema, pleural effusion, or localized abscess, but often failed to provide sufficient anatomical information required for concrete diagnosis and intervention. With recent advancements in CT, rapid diagnosis and proper strategies for pus drainage have become achievable. Additionally, knowledge regarding conservative treatments, including antibiotic use [34], nutritional support [7,22], and cardiorespiratory support, has improved over time. Moreover, the development of endoscopic treatments has allowed the use of less invasive procedures than traditional surgery, which requires general anesthesia; accordingly, the prevalence of cases with postoperative complications has also declined.

In the present study, thoracic surgery was found to be a risk factor for mortality. Considering the predisposing factors for patients with phlegmonous esophagitis, such as old age, diabetes mellitus, alcoholism, and immune suppression, thoracic surgery may be an invasive option, and the surgery itself can lead to serious complications. Furthermore, even though precise localization of lesions can be obtained with CT scans, it may be difficult to distinguish normal tissues for optimal incision and anastomosis sites during esophageal reconstruction surgery. Diffuse, purulent inflammation of phlegmonous esophagitis can spread from the esophagus to the mediastinum or stomach. Although these problems render surgery an inappropriate option, it remains too early to conclude whether endoscopic treatment is superior to surgery. Patients that undergo thoracic surgery may have a more aggravated clinical condition, and the degree of inflammation could differ from that in other patients. There are also some patients for whom endoscopic drainage is unsuitable. Further evaluation and interpretation based on the patient’s context is needed to have an objective view regarding surgical treatment.

With recent advancements in technologies that facilitate rapid diagnosis, an increasing number of patients are being diagnosed with relatively stable conditions. In such cases, appropriate conservative treatment rather than immediate intervention may be worth considering. If a patient does not respond to conservative treatment, endoscopic or surgical intervention should be considered. Additionally, because rapid clinical deterioration is often observed in cases of mortality, close observation is essential.

This systematic review has several limitations. First, the small number of cases limits the ability to generalize the present findings and draw general conclusions regarding this disease. Although we attempted to include articles in several languages, the number of available studies was limited. This led to difficulty in statistical analysis and multivariate risk factor analysis was not possible. Second, many case reports lacked important information, especially regarding continuous variables, such as laboratory data, possibly limiting the strength of the statistical analysis. Finally, as this was a systematic review of case reports, it was based on data reported from various centers, which may have introduced bias and heterogeneity owing to various backgrounds and study periods. However, because of the low incidence of the disease, it is difficult to obtain much larger amounts of data from major institutions.

In conclusion, owing to advancements in CT and endoscopy, phlegmonous esophagitis can now be diagnosed more rapidly and treated using less invasive approaches. This has led to an increasing trend of conservative treatment for patients with early diagnosis and endoscopic treatment for localized abscesses, resulting in favorable in-hospital outcomes. However, the management of diffuse and advanced lesions remains uncertain, and despite aggressive surgical interventions, some cases still show a poor prognosis. Herein, non-survivors demonstrated rapid clinical deterioration, often leading to death within a few days, suggesting a need for intensive treatment. Furthermore, although thoracic surgery was observed to be a significant risk factor for mortality, the study’s limitations prevent us from definitively asserting that endoscopic treatment is superior. Therefore, additional studies are required to establish effective treatment strategies and identify the predictors of patient outcomes.

## Figures and Tables

**Table 1:** Characteristics and clinical findings of phlegmonous esophagitis patients.

Variables	Total (N = 35)	Survivor (N = 32)	Non-Survivor (N = 3)	*p* Value
**Demographics**				
Age	57.0 [48.0–66.0]	58.5 [48.0–65.5]	56.0 [48.0–65.0]	0.930
Male	19/35 (54.3)	16/32 (50.0)	3/3 (100.0)	0.234
**Previous Medical History**				
Hypertension	6/30 (20.0)	6/28 (21.4)	0/2 (0.0)	1.000
Diabetes mellitus	13/30 (43.3)	12/28 (42.9)	1/2 (50.0)	1.000
Alcoholism	5/31 (16.1)	5/29 (17.2)	0/2 (0.0)	1.000
Cancer	2/30 (6.7)	2/28 (7.1)	0/2 (0.0)	1.000
COPD ^(1)^	1/30 (3.3)	1/28 (3.6)	0/2 (0.0)	1.000
Hypothyroidism	1/30 (3.3)	1/28 (3.6)	0/2 (0.0)	1.000
Foreign-body ingestion	8/33 (24.2)	8/31 (25.8)	0/2 (0.0)	1.000
**Clinical Manifestations**				
Onset to admission, days	3.5 [2.3–7.8]	3.5 [2.8–7.8]	3.0 [2.0–4.0]	0.435
Fever	18/32 (56.2)	16/29 (55.2)	2/3 (66.7)	1.000
Gastrointestinal bleeding	5/33 (15.2)	5/30 (16.7)	0/3 (0.0)	1.000
Nausea/Vomiting	8/33 (24.2)	7/30 (23.3)	1/3 (33.3)	1.000
Neck to epigastric pain	32/33 (97.0)	29/30 (96.7)	3/3 (100.0)	1.000
Obstruction/Dysphagia/Foreign-body sensation	19/33 (57.6)	17/30 (56.7)	2/3 (66.7)	1.000
Odynophagia	8/33 (24.2)	8/30 (26.7)	0/3(0.0)	0.560
Oropharyngeal infection	2/33 (6.1)	2/30 (6.7)	0/3 (0.0)	1.000
Upper respiratory infection	5/33 (15.2)	5/30 (16.7)	0/3 (0.0)	1.000
**Vital Signs**				
Body temperatrue, °C	38.3 [37.5–38.9]	38.4 [37.5–38.9]	36.7 [36.7–36.7]	0.130
Systolic blood pressure, mmHg	114.0 [100.0–140.0]	120.0 [111.0–141.5]	99.0 [98.5–99.5]	0.047
Diastolic blood pressure, mmHg	80.0 [69.0–85.0]	80.0 [69.5–85.5]	74.5 [71.8–77.3]	0.619
Heart rate, bmp	98.0 [91.0–113.0]	96.0 [85.5–102.5]	117.0 [115.0–119.0]	0.167
Respiratory rate, bmp	27.0 [23.0–30.0]	27.0 [24.0–28.5]	27.5 [23.3–31.8]	0.769
Fever (over 37.5 °C)	19/25 (76.0)	19/24 (79.2)	0/1 (0.0)	0.240
Tachypnea (respiratory rate over 20/min)	8/10 (80.0)	7/8 (87.5)	1/2 (50.0)	0.378
Hemodynamically unstable	2/13 (15.4)	2/13 (15.4)	0/0	1.000
**Laboratory Results**				
WBC count, /μL	13,100 [7750–22,100]	13,100 [8505–21,550]	12,900 [68,500–18,950]	0.763
Leukocytosis	18/29 (62.1)	17/27 (63.0)	1/2 (50.0)	0.268
Leukopenia	3/29 (10.3)	2/27 (7.4)	1/2 (50.0)	
CRP, mg/dL	19.3 [12.6–31.6]	20.3 [12.6–31.6]	17.1 [17.1–17.1]	0.579
AST, IU/L	58.0 [18.0–76.0]	50.5 [17.8–71.5]	76.0 [76.0–76.0]	0.439
ALT, IU/L	50.5 [24.8–95.8]	34.0 [23.0–102.0]	77.0 [77.0–77.0]	0.602
Cr, mg/dL	0.8 [0.8–1.2]	0.8 [0.8–0.9]	2.10 [1.9–2.4]	0.034
Neutrophil percentage	84.0 [78.5–87.5]	84.0 [79.5–89.0]	61.3 [49.4–73.1]	0.360
Culture (pathogen identified, biopsy)	10/11 (90.9)	10/10 (100.00)	0/1 (0.0)	0.091
Culture (pathogen identified, blood)	5/8 (62.5)	3/6 (50.0)	2/2 (100.0)	0.464
Culture (pathogen identified, pleural fluid)	3/4 (75.0)	3/4 (75.0)	0/0	1.000
Culture (pathogen identified, sputum)	6/7 (85.7)	6/7 (85.7)	0/0	1.000
**Radiologic findings (chest X-ray)**				
Mediastinal widening	4/9 (44.4)	4/9 (44.4)	0/0	1.000
Pleural effusion	5/10 (50.0)	5/10 (50.0)	0/0	1.000
Others ^(2)^	4/9 (44.4)	4/9 (44.4)	0/0	1.000
**Radiologic findings (CT)**				
Abscess	11	11	0	NA
Air bubble	16/18 (88.9)	14/16 (87.5)	2/2 (100.0)	1.000
Diffuse fluid collection/Hypodense lesion (esophagus)	24/24 (100.0)	23/23 (100.0)	1/1 (100.0)	NA
Diffuse wall thickening (esophagus)	27/27 (100.0)	26/26 (100.0)	1/1 (100.0)	NA
Fluid collection/hypodense lesion (other site)	14/14 (100.0)	12/12 (100.0)	2/2 (100.0)	NA
Local wall thickening	13/13 (100.0)	12/12 (100.0)	1/1 (100.0)	NA
Local wall thickening (lower 1/3 esophagus to stomach)	6/6 (100.0)	5/5 (100.0)	1/1 (100.0)	NA
Local wall thickening (upper 2/3 esophagus)	3/3 (100.0)	3/3 (100.0)	0	NA
Mediastinal invasion	10/12 (83.3)	9/11 (81.8)	1/1 (100.0)	1.000
Obvious esophageal mucosal perforation	4/5 (80.0)	4/5 (80.0)	0	1.000
Pleural effusion	14/15 (93.3)	14/14 (100.0)	0/1 (0.0)	0.067
**Esophagogastroduodenoscopy findings**				
Ulcer/Erosion	19/24 (79.2)	19/23 (82.6)	0/1 (0.0)	0.208
Esophageal mucosal perforation	3/24 (12.5)	3/23 (13.0)	0/1 (0.0)	1.000
Esophageal obstruction	17/18 (94.4)	16/17 (94.1)	1/1 (100.0)	1.000
Abscess (total)	6	6	0	NA
Abscess (upper esophagus)	3/4 (75.0)	3/4 (75.0)	0	1.000
Abscess (lower esophagus)	5/6 (83.3)	5/6 (83.3)	0	1.000
Abscess (stomach)	3/4 (75.0)	3/4 (75.0)	0	1.000
**Radiologic findings (Involved site)**				
Pharynx	8/33 (24.2)	8/30 (26.7)	0/3 (0.0)	0.560
Upper esophagus	31/34 (91.2)	29/31 (93.5)	2/3 (66.7)	0.249
Middle esophagus	32/35 (91.4)	30/32 (93.8)	2/3 (66.7)	0.242
Lower esophagus/Gastroesophageal junction	30/35 (85.7)	28/32 (87.5)	2/3 (66.7)	0.380
Stomach	15/35 (42.9)	13/32 (40.6)	2/3 (66.7)	0.565
Lung	18/35 (51.4)	18/32 (56.2)	0/3 (0.0)	0.104
Mediastinum	10/33 (30.3)	9/30 (30.0)	1/3 (33.3)	1.000
**Radiologic findings (other evaluation)**				
Leakage in esophagography	4/9 (44.4)	3/8 (37.5)	1/1 (100.0)	0.444

Data are presented as n, n/N(%), or median [interquartile range]. ^(1)^ Chronic obstructive pulmonary disease. ^(2)^ Including pneumonia, atelectasis, gastric wall air bubble, parenchymal abscess, and paravertebral stripes. AST, aspartate aminotransferase; ALT, alanine transaminase; COPD, chronic obstructive pulmonary disease; Cr, creatinine; CRP, c-reactive protein; CT, computed tomography; WBC, white blood cell.

**Table 2:** Treatment and clinical outcome of patients with phlegmonous esophagitis.

Variables	Total (N = 35)	Survivor (N = 32)	Non-Survivor (N = 3)	*p* Value
**Initial Pus Drainage**				
Interval from admission (days)	2.0 [1.3–5.8]	2.0 [1.0–6.0]	5.0 [5.0–5.00]	0.558
No intervention for drainage	14/35 (40.0)	13/32 (40.6)	1/3 (33.3)	1.000
Endoscopic drainage (ESD)	6/35 (17.1)	6/32 (18.8)	0/3 (0.0)	1.000
Surgical drainage (thoracic)	5/35 (14.3)	3/32 (9.4)	2/3 (66.7)	0.047
**Additional Pus Drainage**				
Interval from the first intervention (days)	11.0 [6.0–20.0]	10.50 [5.5–15.5]	21.0 [21.0–21.0]	0.118
Additional procedure	9/35 (25.7)	8/32 (25.0)	1/3 (33.3)	1.000
Causes for additional procedure (deterioration)	6/8 (75.0)	5/7 (71.4)	1/1 (100.0)	1.000
Causes for additional procedure (residual pus)	2/8 (25.0)	2/7 (28.6)	0/1 (0.0)	1.000
Endoscopic procedure	4/9 (44.4)	4/8 (50.0)	0/1 (0.0)	1.000
Surgical procedure	5/9 (55.6)	4/8 (50.0)	1/1 (100.0)	1.000
**Nutritional Support**				
Enteral feeding via jejunostomy/gastrostomy	6/6 (100.0)	6/6 (100.0)	0	NA
Days from admission to oral intake	36.0 [14.0–74.0]	36.0 [14.0–74.0]	0	NA
**Clinical Outcome**				
Hospital stay (days)	37.0 [17.8–69.0]	52.00 [22.5–74.0]	5.00 [3.0–6.0]	0.006
Complication	22/29 (75.9)	19/26 (73.1)	3/3 (100.0)	0.557
Acute kidney injury	1/22 (4.5)	1/21 (4.8)	0/1 (0.0)	1.000
Gastrointestinal inflammation,/scar	2/23 (8.7)	2/22 (9.1)	0/1 (0.0)	1.000
Disseminated intravascular coagulopathy	4/24 (16.7)	3/22 (13.6)	1/2 (50.0)	0.312
Esophageal stricture	9/24 (37.5)	9/23 (39.1)	0/1 (0.0)	1.000
Septic shock	4/25 (16.0)	3/23 (13.0)	1/2 (50.0)	0.300

**Table 3:** Risk factors univariate analysis for mortality according to clinical presentation and outcome.

Factors	Odds Ratio (95% Confidence Interval)	*p* Value
**History**		
Diabetes mellitus	1.33 (0.08–23.50)	0.84
Age	1.00 (0.92–1.10)	0.93
**Clinical manifestation**		
Nausea/Vomiting	1.64 (0.13–20.90)	0.70
Obstruction/Dysphagia/Foreign-body sensation	1.53 (0.13–18.80)	0.74
**Vital signs**		
Fever (over 37.8 °C)	1.62 (0.13–20.00)	0.70
Hypotension (SBP under 90 mmHg)	1.00 (0.00–inf)	1
Tachypnea (RR over 20)	0.14 (0.00–4.61)	0.27
**Lab and radiologic findings**		
WBC count over 20,000/μL	2.00 (0.11–35.80)	0.64
CRP elevation	0.97 (0.83–1.14)	0.72
Ulcer and/or erosion (EGD)	0.00 (0.00–Inf)	1
Perforation (EGD)	0.00 (0.00–Inf)	1
Radiologic involvement—upper esophagus	0.14 (0.01–2.26)	0.16
Radiologic involvement—middle esophagus	0.13 (0.01–2.18)	0.16
Radiologic involvement—lower esophagus	0.29 (0.24–3.92)	0.35
Radiologic involvement—stomach	2.92 (0.24–35.70)	0.4
Radiologic involvement—lung	0.00 (0.00–Inf)	1
Radiologic involvement—mediastinum	1.17 (0.09–14.60)	0.9
**Pus drainage**		
Pus drainage days from admission	1.03 (0.68–1.54)	0.9
No drain procedure	0.73 (0.06–8.92)	0.81
Pus drainage via thoracic surgery	19.30 (1.33–282.00)	0.03
Pus drainage via endoscopic	0.00 (0.00–Inf)	1

CRP, c-reactive protein; EGD, esophagogastroduodenoscopy; RR, respiratory rate; SBP, systolic blood pressure; WBC, white blood cell.

## Data Availability

The data presented in this study are available on request from the corresponding author.

## Supplementary Materials

The following supporting information can be downloaded at: https://www.mdpi.com/article/10.3390/jcm12227147/s1, Figure S1: PRISMA-P 2020 flow diagram; Table S1: PRISMA-P 2020 Check list; Table S2: Causative pathogen for patients with phlegmonous esophagitis; Table S3: Case abstracts for included studies.
