# Supplementary material for: Optimal Management of Patients with Phlegmonous Esophagitis: A Systematic Review and Meta-Analysis
Source: J Clin Med. 2023 Nov 17;12(22):7147. doi: 10.3390/jcm12227147 (PMC10672419; doi:10.3390/jcm12227147)
Supplement: Supplementary file 1 [file jcm-12-07147-s001.zip › jcm-2687190-supplementary.pdf]

Supplemental Figure S1. PRISMA-P 2020 flow diagram.

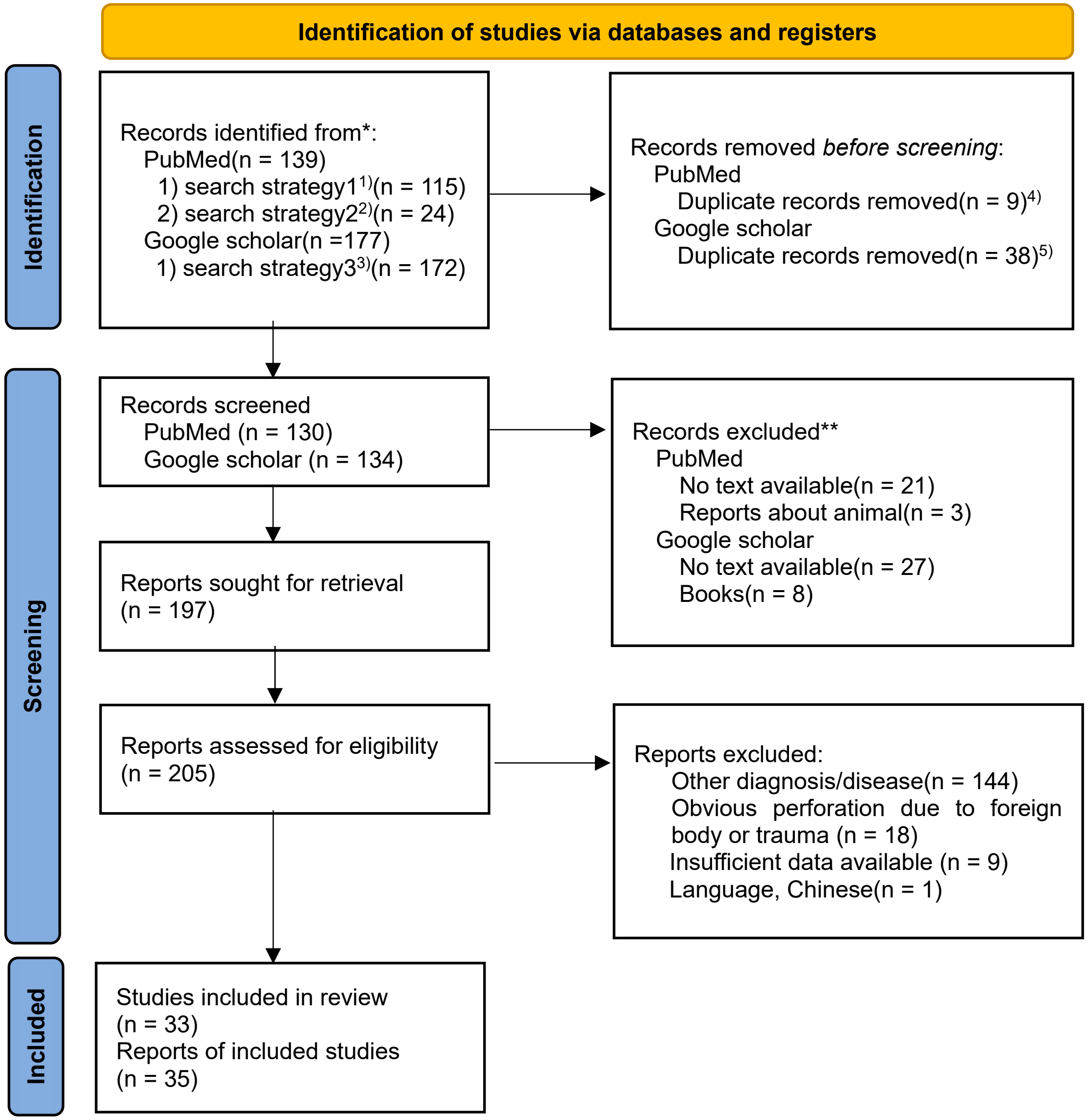

1) Basic search, phlegmonous esophagitis(publication date up to 2023/04/20).

2) Advanced search, “phlegmonous esophagogastritis” OR “phlegmonous gastroesophagitis” OR “phlegmonous enteritis”(publication date up to 2023/04/20).

3) Advanced search, "phlegmonous esophagitis" OR "phlegmonous esophagogastritis" OR "phlegmonous gastroesophagitis".

4) Exclude duplicate results in PubMed search for 1) and 2).

5) Exclude duplicate results within Google Scholar search and those with PubMed.

From: https://www.bmj.com/content/372/bmj.n71

Supplemental Table S1. PRISMA-P 2020 Check list.

Section and Topic	Item #	Checklist item	Location where item is reported
**TITLE**			
Title	1	Identify the report as a systematic review.	Page 1
**ABSTRACT**			
Abstract	2	See the PRISMA 2020 for Abstracts checklist.	Page 1
**INTRODUCTION**			
Rationale	3	Describe the rationale for the review in the context of existing knowledge.	Page 1, 2
Objectives	4	Provide an explicit statement of the objective(s) or question(s) the review addresses.	Page 1, 2
**METHODS**			
Eligibility criteria	5	Specify the inclusion and exclusion criteria for the review and how studies were grouped for the syntheses.	Page 2, 3<br>Figure S1<br>Table S2
Information sources	6	Specify all databases, registers, websites, organisations, reference lists and other sources searched or consulted to identify studies. Specify the date when each source was last searched or consulted.	Page 2, 3<br>Figure S1
Search strategy	7	Present the full search strategies for all databases, registers, and websites, including any filters and limits used.	Page 2, 3<br>Figure S1
Selection process	8	Specify the methods used to decide whether a study met the inclusion criteria of the review, including how many reviewers screened each record and each report retrieved, whether they worked independently, and if applicable, details of automation tools used in the process.	Page 2, 3<br>Figure S1
Data collection process	9	Specify the methods used to collect data from reports, including how many reviewers collected data from each report, whether they worked independently, any processes for obtaining or confirming data from study investigators, and if applicable, details of automation tools used in the process.	Page 2, 3<br>Figure S1
Data items	10a	List and define all outcomes for which data were sought. Specify whether all results that were compatible with each outcome domain in each study were sought (e.g. for all measures, time points, analyses), and if not, the methods used to decide which results to collect.	Page 2, 3
	10b	List and define all other variables for which data were sought (e.g. participant and intervention characteristics, funding sources). Describe any assumptions made about any missing or unclear information.	-
Study risk of bias assessment	11	Specify the methods used to assess risk of bias in the included studies, including details of the tool(s) used, how many reviewers assessed each study and whether they worked independently, and if applicable, details of automation tools used in the process.	-
Effect measures	12	Specify for each outcome the effect measure(s) (e.g. risk ratio, mean difference) used in the synthesis or presentation of results.	Page 3
Synthesis methods	13a	Describe the processes used to decide which studies were eligible for each synthesis (e.g. tabulating the study intervention characteristics and comparing against the planned groups for each synthesis (item #5)).	-
	13b	Describe any methods required to prepare the data for presentation or synthesis, such as handling of missing summary statistics, or data conversions.	Page 3
	13c	Describe any methods used to tabulate or visually display results of individual studies and syntheses.	-
	13d	Describe any methods used to synthesize results and provide a rationale for the choice(s). If meta-analysis was performed, describe the model(s), method(s) to identify the presence and extent of statistical heterogeneity, and software package(s) used.	Page 3
	13e	Describe any methods used to explore possible causes of heterogeneity among study results (e.g. subgroup analysis, meta-regression).	-

Section and Topic	Item #	Checklist item	Location where item is reported
	13f	Describe any sensitivity analyses conducted to assess robustness of the synthesized results.	-
Reporting bias assessment	14	Describe any methods used to assess risk of bias due to missing results in a synthesis (arising from reporting biases).	-
Certainty assessment	15	Describe any methods used to assess certainty (or confidence) in the body of evidence for an outcome.	-
**RESULTS**			
Study selection	16a	Describe the results of the search and selection process, from the number of records identified in the search to the number of studies included in the review, ideally using a flow diagram.	Page 3<br>Figure S1<br>Table S2
	16b	Cite studies that might appear to meet the inclusion criteria, but which were excluded, and explain why they were excluded.	Page 2
Study characteristics	17	Cite each included study and present its characteristics.	Table S2
Risk of bias in studies	18	Present assessments of risk of bias for each included study.	-
Results of individual studies	19	For all outcomes, present, for each study: (a) summary statistics for each group (where appropriate) and (b) an effect estimate and its precision (e.g. confidence/credible interval), ideally using structured tables or plots.	Page 3, 4, 5<br>Table 1, 2
Results of syntheses	20a	For each synthesis, briefly summarise the characteristics and risk of bias among contributing studies.	-
	20b	Present results of all statistical syntheses conducted. If meta-analysis was done, present for each the summary estimate and its precision (e.g. confidence/credible interval) and measures of statistical heterogeneity. If comparing groups, describe the direction of the effect.	Page 3, 4, 5, 6, 7, 8<br>Table 1, 2, 3
	20c	Present results of all investigations of possible causes of heterogeneity among study results.	-
	20d	Present results of all sensitivity analyses conducted to assess the robustness of the synthesized results.	-
Reporting biases	21	Present assessments of risk of bias due to missing results (arising from reporting biases) for each synthesis assessed.	-
Certainty of evidence	22	Present assessments of certainty (or confidence) in the body of evidence for each outcome assessed.	-
**DISCUSSION**			
Discussion	23a	Provide a general interpretation of the results in the context of other evidence.	Page 9, 10
	23b	Discuss any limitations of the evidence included in the review.	Page 10
	23c	Discuss any limitations of the review processes used.	Page 10
	23d	Discuss implications of the results for practice, policy, and future research.	Page 10
**OTHER INFORMATION**			
Registration and protocol	24a	Provide registration information for the review, including register name and registration number, or state that the review was not registered.	-
	24b	Indicate where the review protocol can be accessed, or state that a protocol was not prepared.	-
	24c	Describe and explain any amendments to information provided at registration or in the protocol.	-
Support	25	Describe sources of financial or non-financial support for the review, and the role of the funders or sponsors in the review.	Page 10

Section and Topic	Item #	Checklist item	Location where item is reported
Competing interests	26	Declare any competing interests of review authors.	Page 10
Availability of data, code and other materials	27	Report which of the following are publicly available and where they can be found: template data collection forms; data extracted from included studies; data used for all analyses; analytic code; any other materials used in the review.	Page 10

From: https://www.bmj.com/content/372/bmj.n71

Supplemental Table S2. Systhesis Without Meta-analysis (SWiM) reporting check list

**SWiM is intended to complement and be used as an extension to PRISMA**			
**SWiM reporting item**	**Item description**	**Page in manuscript where item is reported**	**Other***
Methods			
**1** Grouping studies for synthesis	1a) Provide a description of, and rationale for, the groups used in the synthesis (e.g., groupings of populations, interventions, outcomes, study design)	Page 1, 2	
	1b) Detail and provide rationale for any changes made subsequent to the protocol in the groups used in the synthesis	Page 2, 3	
**2** Describe the standardised metric and transformation methods used	Describe the standardised metric for each outcome. Explain why the metric(s) was chosen, and describe any methods used to transform the intervention effects, as reported in the study, to the standardised metric, citing any methodological guidance consulted	-	
**3** Describe the synthesis methods	Describe and justify the methods used to synthesise the effects for each outcome when it was not possible to undertake a meta-analysis of effect estimates	Page 2, 3	
**4** Criteria used to prioritise results for summary and synthesis	Where applicable, provide the criteria used, with supporting justification, to select the particular studies, or a particular study, for the main synthesis or to draw conclusions from the synthesis (e.g., based on study design, risk of bias assessments, directness in relation to the review question)	Page 2, 3<br><br>Figure S1	
**SWiM reporting item**	**Item description**	**Page in manuscript where item is reported**	**Other***
**5** Investigation of heterogeneity in reported effects	State the method(s) used to examine heterogeneity in reported effects when it was not possible to undertake a meta-analysis of effect estimates and its extensions to investigate heterogeneity	-	
**6** Certainty of evidence	Describe the methods used to assess certainty of the synthesis findings	Page 3	
**7** Data presentation methods	Describe the graphical and tabular methods used to present the effects (e.g., tables, forest plots, harvest plots).<br>Specify key study characteristics (e.g., study design, risk of bias) used to order the studies, in the text and any tables or graphs, clearly referencing the studies included	Page 3	

Results			
**8** Reporting results	For each comparison and outcome, provide a description of the synthesised findings, and the c ertainty of the findings. Describe the result in language that is consistent with the question the synthesis addresses, and indicate which studies contribute to the synthesis	Page 3, 4, 5, 6, 7, 8	
Discussion			
**9** Limitations of the synt hesis	Report the limitations of the synthesis methods used and/or the groupings used in the synthesi s, and how these affect the conclusions that can be drawn in relation to the original review qu estion	Page 9, 10	

https://www.bmj.com/content/368/bmj.l6890

Supplemental Table S3. Case abstracts for included studies and causative pathogen for each patient

Authors	Number of cases	year of report	Case abstract	References	Causative pathogen	
					Specimen	Culture results
Kim, M.J., et al.	1	2022	A 67-year-old man with underlying DM experienced neck pain. The patient underwent endoscopy and CT and was diagnosed with acute phlegmonous esophagitis. Empirical antibiotics and endoscopic drainage were administered, and the patient was discharged with esophageal stricture.	doi: 10.4166/kjg.2022.098.	Blood	None
Yun, S.M., et al.	1	2022	A 76-year-old woman experienced neck pain, fever, and foreign body sensation. The patient underwent endoscopy and CT and was diagnosed with acute phlegmonous esophagitis with partial tear of esophageal wall. Antibiotics and endoscopic drainage were administered, and the patient discharged with esophageal stricture.	doi: 10.3390/medicina58070864.	sputum	Streptococcus viridans groups
Zhang, Z., et al.	1	2022	A 40-year-old man was transferred due to worsening of neck pain after thoracotomy for acute phlegmonous esophagitis. The patient underwent CT and contrast esophagography and was diagnosed with acute phlegmonous esophagitis with mediastinal abscess and pharyngeal abscess. Empirical antibiotics and thoracic drainage were administered, but the patient died with sudden cardiac arrest.	doi: 10.21037/qims-21-573	-	-
Santos, A.L., et al.	1	2022	A 48-year-old woman with a history of alcohol abuse and major depressive disorder was diagnosed with phlegmonous esophagogastritis, mediastinitis, empyema, and septic shock following endoscopy and CT scans. The patient received ICU care, empirical antibiotics, and repeated endoscopic drainage, resulting in clinical improvement and discharge.	doi: 10.1159/000521485.	pus	Escherichia coli<br>Veillonella atypia<br>Candida albicans
Saito Y et al.	1	2021	A 61-year-old woman with a history of HTN and glaucoma was diagnosed with phlegmonous esophagitis, deep neck infection and empyema following CT scans. Antibiotics, thoracic, cervical drainage and endoscopic false lumen dissection were administered, and the patient was discharged.	doi: 10.1016/j.athoracsur.2020.08.101	pus	Parvimonas micra
Vates, Gomez, R., et al.	1	2020	A 43-year-old man with fever and retrosternal chest pain was diagnosed with acute phlegmonous esophagitis and pleural effusion after CT scan. Antibiotics and conservative treatment were applied and the patient had good clinical outcome.	doi: 10.17235/reed.2020.6742/2019.	pleural fluid	None
Kim J.W., et al.	2	2019	(1) A 65-year-old man with a history of DM and COPD was transferred due to uncontrolled esophageal abscess. The patient underwent CT scan and was diagnosed with acute phlegmonous esophagitis with empyema. Antibiotics and endoscopic drainage were administered and the patient was discharged without complications.<br>(2) A 57-year-old woman was transferred with hematemesis and nausea after an EGD at another hospital. Following CT and endoscopy, the patient was diagnosed with phlegmonous esophagitis and mediastinitis. Antibiotics and natural intraluminal drainage were administered and the patient was discharged without complications.	doi: 10.5090/kjtcs.2019.52.3.165	-	-
Woo, W.G., et al.	1	2017	A 67-year-old woman with a history of DM and hypothyroidism presented epigastric pain and was diagnosed with acute phlegmonous esophagitis with multiple esophageal perforation and pleural effusion following endoscopy and CT. Antibiotics and intraluminal natural	doi: 10.5090/kjtcs.2017.50.6.453	Blood, Sputum	None

			drainage were administered, the patient was discharged with esophageal stricture.			
Huang, Y.C., et al.	1	2017	A 60-year-old woman with a history of DM presented fever, chest pain and dysphagia. Follow up CT and endoscopy revealed acute phlegmonous esophagogastritis with mediastinitis, hypopharyngeal abscess, and empyema, which was treated with antibiotics and surgery. The patient underwent esophageal reconstruction after clinical improvement.	doi: 10.12659/ajcr.902180.	sputum	Pseudomonas aeruginosa
					pleural fluid	Klebsiealla pneumoniae Pseudomonas aeruginosa
Hashimoto, R., et al.	1	2016	A 72-year-old woman with a history of schizophrenia presented fever, dysphagia, mouth erythema and hematemesis. The patient underwent CT and endoscopy, and was diagnosed erysipelas followed by phlegmonous esophagogastritis. Antibiotics and conservative management were administered, the patient was discharged without complication.	doi: 10.1053/j.gastro.2016.06.011.	facial skin	a-streptococcus spp.
					gastric biopsy	a-streptococcus spp.
Inoue, M., et al.	1	2016	A 54-year-old man with a history of DM, panic disorder, and alcohol abuse showed neck pain, odynophagia, dysphagia, and dyspnea. The patient was diagnosed with phlegmonous esophagitis, secondary to peritonsillar abscess following laryngoscopy and CT. Antibiotics and tonsillar abscess incision and drainage were administered, the patient was discharged with clinical improvement.	doi:10.3950/jibiinkoka.119.962	pus	Streptococcus constellatus
Matsumoto, H., et al.	1	2015	A 74-year-old man with myelofibrosis and multiple myeloma had acute epigastric pain and nausea. The patient was diagnosed with acute phlegmonous esophagogastritis including duodenal invasion. Despite antibiotics treatment, the patient died 14 hours after arrival due to multi-organ failure.	doi: 10.3748/wjg.v21.i12.3741.	blood(PCR)	Bacillus thuringiensis
					gastric fluid	G(+) cocci G(+) rods G(-) rods Bacillus thuringiensis(PCR)
Karimata, H., et al.	1	2014	A 47-year-old woman who underwent chemotherapy presented epigastric pain, dyspnea, nausea, and vomiting. The patient was diagnosed with acute phlegmonous esophagitis following CT scan. The patient recovered from multi-organ failure with appropriate management, but further treatments including amputation and pneumatic dilatation of esophagus were required.	doi: 10.1007/s00595-013-0536-2.	pleural fluid	Streptococcus milleri
Chang, P.C., et al.	1	2012	A 57-year-old woman with a history of HTN showed dysphagia and odynophagia and was diagnosed with acute phlegmonous esophagitis following endoscopy and CT scan. Antibiotics, cervicotomy were initially administered. In the process of treatment, NG tube insertion caused esophageal perforation and additional endoscopic drainage was applied. The patient discharged without severe complication.	doi: 10.1016/j.ejcts.2011.06.027.	pus	Klebsiella pneumoniae
Kim, H.S., et al.	1	2010	A 48-year-old man with a history of DM and alcoholism presented neck pain, dysphagia, and dyspnea. The patient was diagnosed with acute phlegmonous esophagogastritis and underwent repeated thoracotomy and antibiotics treatment. The patient was discharged with clinical improvement.	doi: 10.3346/jkms.2010.25.10.1532.	blood	Klebsiella pneumoniae
					sputum	Klebsiella pneumoniae
					pleural fluid	Klebsiella pneuminiae

Shiozawa, K., et al.	1	2009	A 62-year-old man presented with sore throat and chest pain was diagnosed with acute phlegmonous esophagogastritis following CT and endoscopy. Antibiotics treatment, tracheostomy, and hypopharyngeal abscess drainage were performed and the patient was discharged without complications.	doi:10.11405/nisshoshi.106.370	-	-
Imai, A., et al.	1	2005	A 73-year-old woman presented with fever and neck pain was diagnosed with acute phlegmonous esophagogastritis following CT. Multiple perforations were found in following endoscopy and the patient underwent antibiotics treatment. The patient was discharged with clinical improvement.	doi:10.11405/nisshoshi.102.1534	esophageal ulcer biopsy	a-streptococcus spp.
Yun, C.H., et al.	1	2005	A 63-year-old woman with a history of HTN and type 2 DM presented sore throat, chest pain, and odynophagia. The patient was diagnosed with acute phlegmonous esophagitis and was clinically improved after antibiotics and DM control.	doi: 10.1007/s00330-005-2842-6.	sputum	G(+) bacilli
Jung, C., et al.	1	2003	A 52-year-old man presented with fever, dysphagia and sore throat and was diagnosed with acute phlegmonous esophagogastritis with intramural abscess. There were multiple mucosal ulcers in follow up endoscopy and antibiotics and conservative care were applied. The patient was discharged with clinical improvement.	doi: 10.2214/ajr.180.3.1800862.	sputum	G(+) bacilli
Hsu, C.Y., et al.	1	1996	A 42-year-old man was transferred due to fever, epigastraliga and odynophagia. Initially, antibiotics and conservative treatment was administered. However, total gastrectomy with duodenostomy, transhiatal near total esphagectomy cervical esophagostomy was required due to clinical worsening. The patient was discharged after several anastomosis.	PMID : 8909573	gastrectomy biopsy	bacterial clump
Wakayama, T., et al.	1	1994	A 31-year-old man with symptoms of fever, epigastric pain, and vomiting. The patient was diagnosed with phlegmonous gastritis with esophageal involvement. Antibiotics, total gastrectomy, subtotal esophagectomy, and RnY anastomosis were performed. The patient was discharged without complication.	PMID: 8172161	blood	Bacteroides spp.
					gastric fluid	Enterobacter cloacae Klebsiella pneumoniae
Mann, N.S., et al.	1	1978	A 62-year-old man with underlying diverticulum presented fever, retrosternal pain and odynophagia. The patient was diagnosed with phlegmonous esophagitis and lung abscess following CT, endoscopy and esophagogram. Antibiotics and conservative treatment were administered and the patient was discharged with clinical improvement.	PMID: 105632	blood	None
					sputum	Mixed flora
Kawakubo, H., et al.	1	2002	A 51-year-old man presented with fever, sore throat, vomiting, and melena and was diagnosed with acute phlegmonous esophagogastritis following CT and endoscopy. The patient was discharged without complication after broad-spectrum antibiotics and conservative treatment.	doi: 10.11280/gee1973b.44.990	gastric fluid	None
Nishiya, S., et al.	1	2007	A 43-year-old man presented with fever, jaundice and odynophagia and was diagnosed with DIC and acute phlegmonous esophagogastritis. Despite conservative treatment, the symptoms were not improved and esophageal bypass surgery were conducted. The patient was discharged with clinical improvement.	doi :10.5833/jjgs.40.1655	blood	Klebsiella pneumoniae

FURUCHI, K., et al.	1	1998	A 49-year-old man with a history of tonsillitis presented chest pain and upper abdominal pain and was diagnosed with peritonitis due to acute esophagogastric phlegmon. The patient underwent abdominal surgical drainage, and was discharged with clinical improvement.	doi :10.3919/jjsa.59.112	-	-
Chang Ryoul Lee, et al.	1	2000	A 21-year-old healthy man presented fever and epigastric pain and was diagnosed with acute phlegmonous esophagitis following CT, endoscopy and esophagogram. Antibiotics and thoracotomy were applied and the patient was discharged without complications.	Korean Journal of Gastrointestinal Endoscopy 2000;20(2):119-123	pus	Staphylococcus aureus
Ko, R., J. Baby and K.P. Valalan	1	2019	A 56-year-old man with a history of DM was transferred due to clinical worsening was diagnosed with acute phlegmonous esophagogastritis following CT and endoscopy. Antibiotics treatment and explorative thoracotomy were performed but the patient died.	doi: 10.14309/crj.0000000000000042.	blood<br>gastric mucosa biopsy	Klebsiella pneumoniae<br>None
Ahn et al.	1	2020	A 76-year-old woman with a history of HTN showed neck pain, vomiting and fever. The patient was diagnosed with acute phlegmonous esophagitis with abscess and antibiotics treatment and endoscopic internal drainage were performed. The patient's clinical course has improved.	Korean Journal of Internal Medicine Abstracts for annual conference 2020; S-057	-	-
Minji, Kim, et al.	1	2021	A 62-year-old man was transferred uncontrolled abscess and mucosal defect. The patient underwent CT and endoscopy and was diagnosed with phlegmonous esophagitis. Antibiotics and endoscopic intraluminal drainage were administered. After discharge, repeated esophageal dilatations were needed due to esophageal stricture.	doi: 10.4166/kjg.2022.098	-	-
Sang hoon Lee, et al.	1	2018	A 60-year-old woman presented with sore throat, chest pain, dyspnea and dysphagia. CT and endoscopy were performed and the patient was diagnosed with retropharyngeal abscess with acute phlegmonous esophagitis. Antibiotics and arytenoid abscess drainage were administered. The patient was discharged without complications.	doi: 10.35420/jcohns.2018.29.1.119	-	-
I, H.S., et al.	1	2007	A 69-year-old man with a history of DM and HTN presented fever and neck pain and diagnosed with phlegmonous esophagitis with mediastinits. Antibiotics and thoracotomy were administered and the patient was discharged without complication.	Korean J Thorac Cardiovasc Surg 2007; 40(10): 711-714	-	-
Taehoon Kim, et al.	1	2019	A 71-year-old woman with underlying DM and steroid treatment presented chest pain, dysphagia, and odynophagia. The patient underwent CT and endoscopy, and was diagnosed with acute phlegmonous esophagogastritis. Antibiotics and supportive care were administered and the patient discharged without complications.	Doi: 10.4166/kjg.2019.73.4.239	-	-
Yoon, J.W., et al.	2	2015	(1) A 26-year-old woman with DM present sore throat, neck pain and hoarseness. The patient underwent CT and endoscopy was diagnosed with acute phlegmonous esophagogastritis. Antibiotics and conservative treatment were administered and the patient discharged without complications.	doi : 10.4093/jkd.2015.16.2.153	gastroesophageal junction biopsy	Klebsiella pneumoniae

			(2) A 56-year-old woman with a history of breast cancer and DM presented sore throat and neck pain. The patient underwent CT and was diagnosed with acute phlegmonous esophagogastritis. Antibiotics treatment, cervical incision, and drainage were performed. Esophagectomy and reconstruction surgery were additionally needed due to esophageal perforation. The patient was discharged with clinical improvement.		pus	Klebsiella pneumoniae Streptococcus viridans
